# Total knee arthroplasty improves sports activity and the patient-reported functional outcome at mid-term follow-up

**DOI:** 10.1007/s00167-022-07025-z

**Published:** 2022-06-11

**Authors:** Amit Meena, Christian Hoser, Elisabeth Abermann, Caroline Hepperger, Akshya Raj, Christian Fink

**Affiliations:** 1 Gelenkpunkt - Sports and Joint Surgery, Olympiastraße 39, 6020 Innsbruck, Austria; 2 Research Unit for Orthopaedic Sports Medicine and Injury Prevention (OSMI), Private University for Health Sciences, Medical Informatics and Technology, Innsbruck, Austria; 3 Central Institute of Orthopaedics, Vardhman Mahavir Medical College and Safdarjung Hospital, New Delhi, 110029 India

**Keywords:** TKA, Total knee arthroplasty, Total knee replacement, TKR, Functional outcome, Sports activity, Survivorship

## Abstract

**Purpose:**

The purpose of this study was to assess (1) sports participation and preference for the type of sports activity after TKA, (2) mid-term functional outcome and activity level, (3) correlation of different age groups with activity level and functional outcomes, and (4) mid-term survivorship of the prosthesis.

**Methods:**

A retrospective review of prospectively collected data was performed. 182 patients were included who underwent primary TKA between January 2010 and December 2016. Inclusion criteria were symptomatic knee osteoarthritis, age 50–90 years, and with a minimum of 5-year follow-up after TKA. Patients with rheumatoid arthritis and revision TKA were excluded. Sports participation and sports preference, Oxford Knee Score (OKS), Tegner Activity Level, and Visual Analogue Scale (VAS) for pain were recorded pre- and postoperatively at 6 months, 1 year, 2 years, and 5 years. The patient cohort was subdivided according to age groups; activity levels, patient-reported outcomes, and improvement in knee pain were correlated with these age groups. Kaplan–Meier curves were used to investigate survivorship at a minimum of 5 years.

**Results:**

The mean age of the cohort was 75.6 ± 7.2 years (range 52–89). Significant improvement was noted in sports participation (*p* < 0.003). After TKA, there was no change in the preference for sports and none of the patients had to discontinue their sporting activity. OKS improved significantly at all follow-up time points compared to the preoperative score (*p* < .0001). Patients' sports and physical activity improved significantly at 1 year compared to the preoperative activity level (*p* < 0.001). Although the Tegner activity level improved over time, this improvement was not significant (NS), while it was significantly higher in males than in females (*p* < 0.004). Significant improvement was found in the VAS for pain at all follow-up time points compared to the preoperative score (*p* < .0001). Survivorship was found to be 100% at a 5-year follow-up.

**Conclusion:**

After TKA, patients can be able to return to sporting activity or even perform better than before surgery. Maximum improvement was noted in the first post-operative year. The male and younger groups perform better than the female and older groups. Sports and physical activity do not negatively impact survivorship of the knee prosthesis at mid-term follow-up and all patients are encouraged to take up sports participation after their TKA.

**Level of evidence:**

Level 3.

## Introduction

Total knee arthroplasty (TKA) is the treatment of choice for end-stage osteoarthritis (OA) where more conservative options have failed [21]. With continuously evolving implant technology, surgical technique, and procedural safety over the past decade, patients have high expectations for the outcome of knee arthroplasty. Therefore, the success of knee arthroplasty is more and more evaluated by the ability to return to sporting and recreational activities with excellent survivorship of the prosthesis [20].

Sports activities enhance muscle strength, coordination, balance, and bone density, and therefore reduce the risk of implant loosening [5, 12]. Moreover, sports activities provide opportunities for social interaction, developing relationships, and contributing to the overall sense of successful aging [18]. Therefore, patients frequently ask the treating surgeon, whether they will be able to return to sporting activity or even perform better than before surgery [14]. On the other hand, surgeons are cautious and forbid high-impact sports and physical activities to prevent premature failure due to prosthetic wear, loosening, and revision [4]. Therefore, potential negative consequences of sporting activities on TKA should be considered in balance with the beneficial effects. Considering this challenge, surgeons need to provide detailed information to the patient and set realistic goals for a post-operative functional outcome [8, 13]. Studies have shown that activity level and functional outcome improved after TKA, but these studies had a short-term follow-up and they did not evaluate survivorship of TKA [9, 16]. On the other hand, one study found that the rate of sporting activity decreased after TKA, even if surgery improved functional outcomes and patients’ activity levels [3].

Therefore, this study aimed to analyze (1) sports participation and preference for the type of sports activity after TKA, (2) mid-term functional outcome and activity level, (3) correlation of different age groups with activity level and functional outcomes, and (4) mid-term survivorship of the prosthesis. The hypothesis was that (1) TKA will have a positive effect on sports participation and activity levels with significant improvement in functional outcomes, (2) younger age groups will have a better functional outcome and activity levels, and (3) prosthesis survivorship will not be deteriorated by sports activities.

## Materials and methods

A retrospective review of prospectively collected data was performed to identify patients who underwent primary total knee arthroplasty. Patients were included in the study if they fulfilled the following inclusion criteria: diagnosis of primary symptomatic knee osteoarthritis (Kellgren and Lawrence grade 3 or 4), age 50–90 years, and had a minimum of 5-year follow-up after TKA. The exclusion criteria were: rheumatoid arthritis, revision TKA, and conditions that might interfere with the standard post-operative rehabilitation protocol.

For this consecutive retrospective series, 204 patients were identified who underwent primary TKA between January 2010 and December 2016. Twenty-two patients did not meet the inclusion criteria (*n* = 4 were out of age limit; *n* = 12 did not give their written informed consent; *n* = 6 lost to follow-up. Since lost to follow-up was small (*n* = 6), therefore, missing values imputation was not considered for this. Finally, a total of 182 patients were included in this study. All the surgeries were performed by two senior surgeons. The medial parapatellar approach and cemented cruciate-retaining total knee prosthesis (NexGen CR, Zimmer Inc.) were used. All patients were given a standardized post-operative rehabilitation program that consisted of a four-point gait pattern within the first 2 weeks after surgery. The patients were advised to use crutches for initial 4 weeks. For the next 8 weeks, low-impact physical activities such as walking, swimming, and static cycling were recommended. After 12 weeks, hiking, cycling, Nordic walking, golf, and weight training exercises were allowed. According to the progress of the individual subject, further sports activities such as mountain biking, skiing, and tennis were allowed at 5 to 6 months.

Patients were evaluated pre-and postoperatively at 6 months, 1 year, 2 years, and 5 years for sports participation and sports preference, patient-reported outcomes, activity levels, and improvement in knee pain. A preoperative questionnaire evaluated the patients’ condition within the last 4 weeks before surgery rather than immediately preoperatively. Sports participation and the type of sports most frequently performed after surgery were evaluated by a direct question. The top three sports preferences in the winter and summer sessions were noted. Tegner Activity level Scale, ranging from 0 (= sick leave or disability pension because of knee problems) to 10 (= competitive sports— national elite), measured the activity level. For patient-reported outcomes, Oxford Knee Score (OKS) was used. It is a self-reported questionnaire that evaluates patients’ perceptions following TKA. OKS consist of 12 questions covering pain, stiffness, and function parameters, scored from 0 to 48, with higher scores indicating better patient-reported outcomes. The pain was measured with the Visual Analogue Scale (VAS) for pain. Patients specified their perception of the pain on a scale from 0 (= no pain) to 10 (= severe pain).

The study was conducted at Gelenkpunkt—Sports and Joint surgery and approved by the ethics committee of the Medical University of Innsbruck (AN2016-0117).

### Statistical analysis

The sample size was calculated based on a previous study by Hepperger et al. [9]. They observed proportion of patients who participated in sports preoperatively and postoperatively at 6, 12, and 24 months was 79%, 82%, 82%, and 83%, respectively. Taking this value as reference, the minimum required sample size with a 6% margin of error and 5% level of significance was 178 patients. To reduce the margin of error, the total sample size taken was 182 patients.

To calculate sample size, this formula was used:

$$n\, \ge \,\left( {p\left( {1 - p} \right)} \right)/\left( {{\text{ME}}/z_{\alpha } } \right)^{2}$$

where *Z*_*α*_ is the value of *Z* at a two-sided alpha error of 5% (i.e., 1.96).

ME is a margin of error (6%).

*p* is the proportion of patients who participated in sports at various time intervals.

The presentation of the categorical variables was done in the form of numbers and percentages (%). On the other hand, the quantitative data with normal distribution were presented as the means ± SD and the data with non-normal distribution as median with 25th and 75th percentiles (interquartile range). The data normality was checked using the Kolmogorov–Smirnov test. In the cases the data were not normal, non-parametric tests were used. The comparison of the variables which were quantitative and not normally distributed in nature was performed using the Mann–Whitney test (for two groups) and the Kruskal–Wallis test (for more than two groups). For outcome comparison between age groups, the Kruskal–Wallis test along with a Post Hoc analysis by Dunn’s multiple pairwise comparison test was carried out. Friedman test was used for comparison across follow-up along with post hoc comparison. All the qualitative variables were analyzed using Fisher’s exact test. The patient cohort was subdivided according to age groups; activity levels, patient-reported outcomes and improvement in knee pain were correlated with these age groups. Additionally, Kaplan–Meier curve analysis was planned for the survivorship, with failure defined as a revision of any component for any reason. The data entry was done in the Microsoft EXCEL spreadsheet and the final analysis was done with the use of Statistical Package for Social Sciences (SPSS) software, IBM manufacturer, Chicago, USA, version 21.0. For statistical significance, a p value of less than 0.05 was considered statistically significant.

## Results

Out of 182 patients, 97 (53.3%) were female and 85 (46.7%) were male. The mean age at the time of surgery was 75.6 ± 7.2 years (range, 52–89). Patients were divided into four different age groups (Table1).

**Table 1:** Demographic data of the study population

Variable	Results
Total patients	182
Gender	Male = 85; Female = 97
Mean age	75.6 ± 7.2 (range 52–89)
Patient number in the age group of 50–59	5 (2.7%)
Patient number in the age group of 60–69	31(17.0%)
Patient number in the age group of 70–79	86 (47.2%)
Patient number in the age group of 80–89	60 (32.9%)

### Sports participation and preference for sports activity

Significant improvement was noted in sports participation (*p* < 0.003) (Table 2). After TKA, the most common sports practiced were, hiking, cycling, and swimming in order of decreasing frequency in summer; and in winter, the most commonly practiced sports were skiing followed by cross-country skiing and then hiking, over all the age groups over the entire follow-up period. The preference for sporting activity had not changed from preoperative status and all the patients continued to practice their preferred sports with none of the patients having to discontinue.

**Table 2:** Comparison of sports participation of study subjects across follow-up at preoperatively and postoperatively 6 months, 1 year, 2 years, and 5 years

Sports participation	Pre-operative	6 months	12 months	24 months	60 months	*P* value
	(*n*)	(*n*)	(*n*)	(*n*)	(*n*)	
Occasionally	44 (24.2%)	23 (12.6%)	21 (11.5%)	19 (10.4%)	19 (10.4%)	0.003^a^
1 to 2 times a week	34 (18.7%)	27 (14.8%)	23 (12.6%)	25 (13.7%)	23 (12.6%)	
3 to 5 times a week	85 (46.7%)	102 (56.0%)	105 (57.7%)	107 (58.8%)	104 (57.1%)	
More than 5 times a week	19 (10.4%)	30 (16.5%)	33 (18.1%)	31 (17.0%)	36 (19.8%)	

^a^Chi-square test

(*n*) Number of patients

### Patient-reported outcomes

**Fig. 1:**
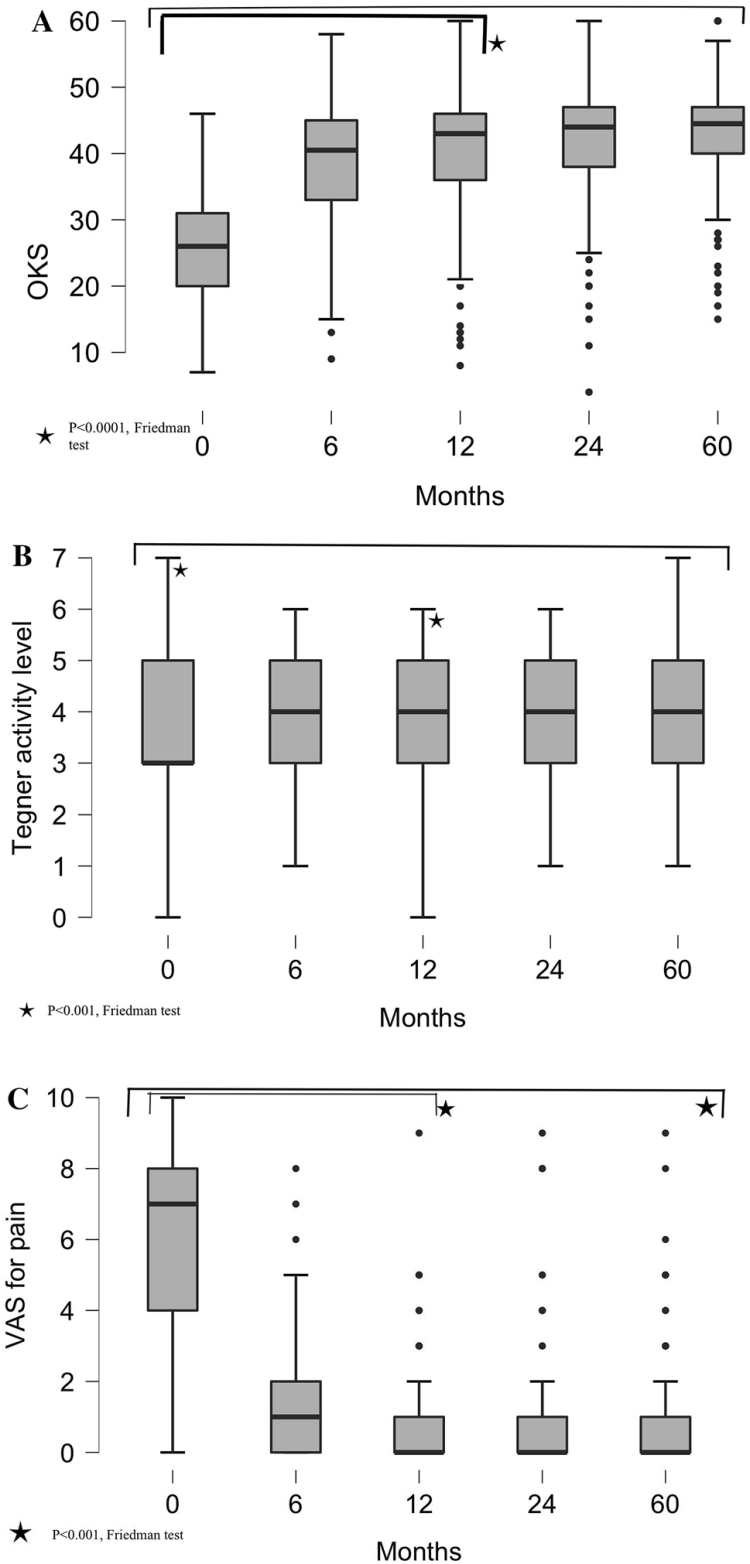
Whisker plots at preoperatively and postoperatively 6 months, 1-year, 2-year, 5-year follow-up for **A** OKS, **B** Tegner activity level, and **C** VAS for pain scores. Asterisks mark shows the statistically significant comparisons. Post-operative OKS, Tegner, and VAS for pain improved significantly compared to the preoperative score

**Fig. 2:**
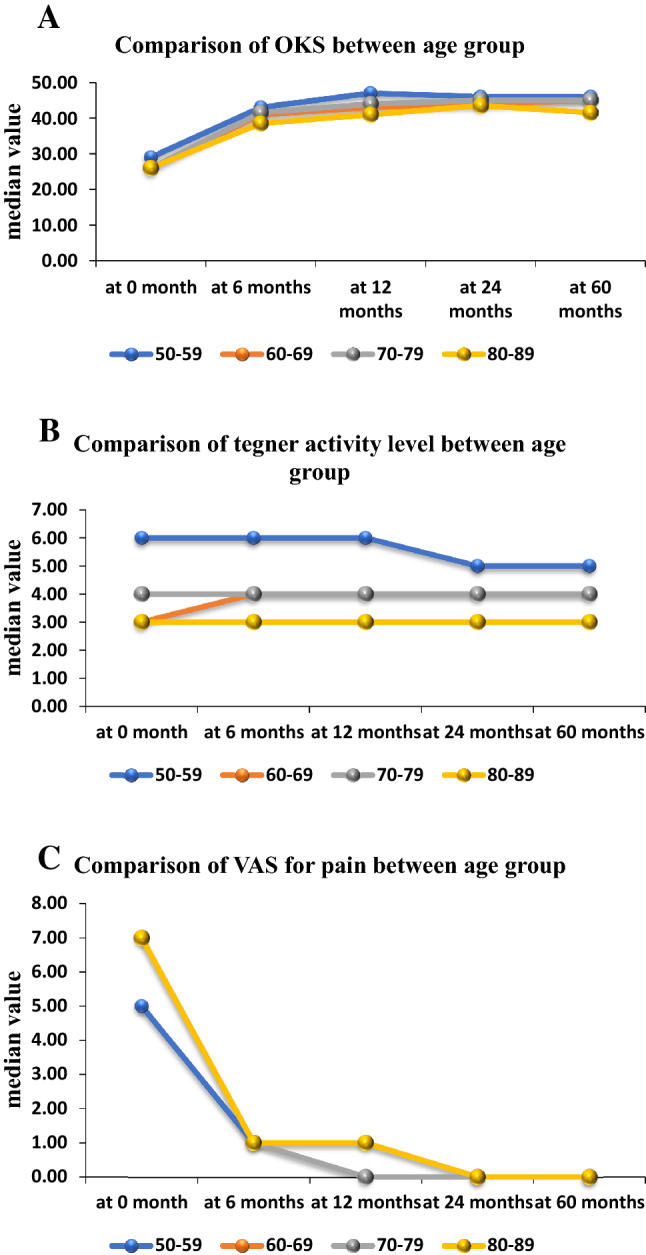
Comparison of the **A** OKS, **B** Tegner activity level, and **C** VAS for pain scores between 5 age subgroups at preoperatively and postoperatively 6 months, 1-year, 2-year, and 5-year follow-up. Post-operative OKS, Tegner, and VAS for pain improved significantly compared to the preoperative score

**Fig. 3:**
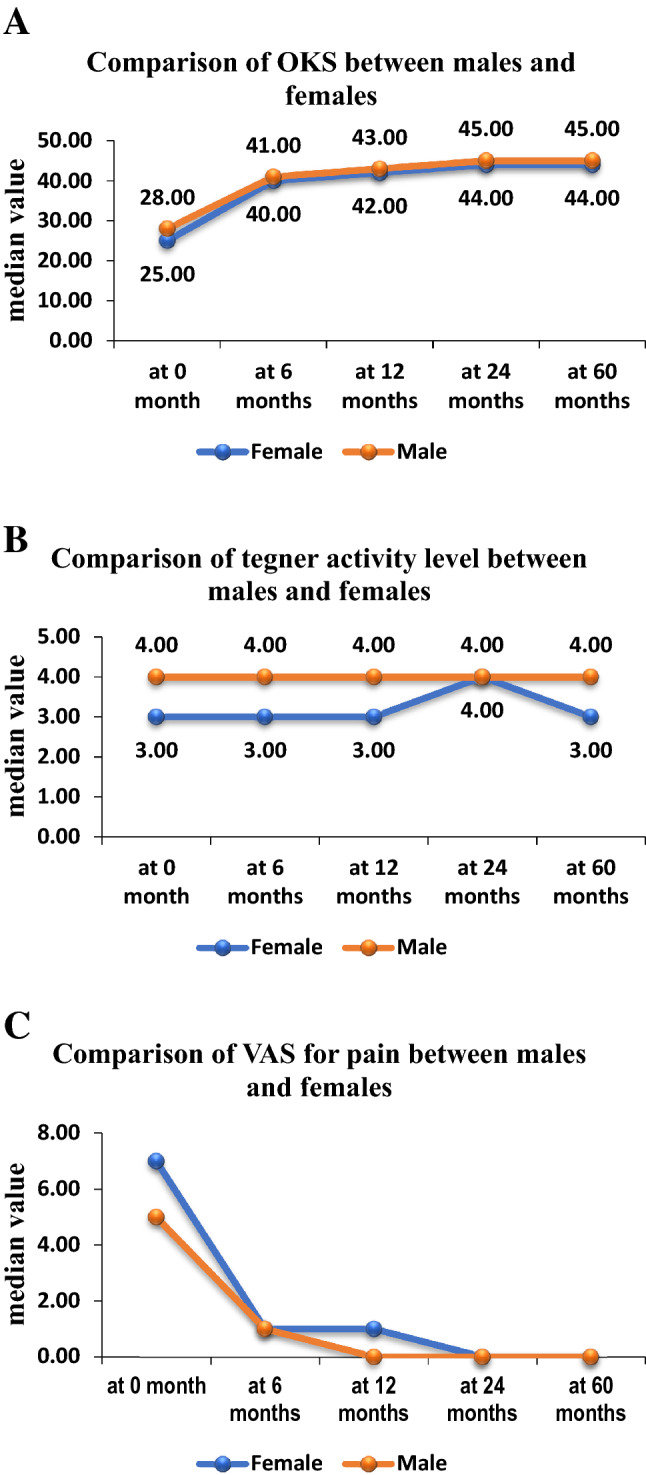
Comparison of the **A** OKS, **B** Tegner activity level, and **C** VAS for pain scores between male and female patients at preoperatively and postoperatively 6 months, 1-year, 2-year, and 5-year follow-up. Post-operative OKS and VAS for pain between male and female patients were non-significant. Tegner activity level is significantly higher in males

The Oxford knee score (OKS) improved significantly at all follow-up time points compared to the preoperative score (*p* < 0.0001). Comparison of OKS was performed at different follow-up periods and this did not improve significantly when comparing the scores at 1 year, 2 years, and 5 years (Table 3 and Fig. 1A) (NS). OKS was also analyzed for different age groups at different follow-up periods and no significant difference was found among the age groups (Table 4 and Fig. 2A) (NS). Also, no significant difference was observed in the post-operative OKS between male and female patients (Fig. 3A) (NS).

**Table 3:** Comparison of OKS, tegner activity level, and VAS for pain of study subjects across follow-up at preoperatively and postoperatively 6 months, 1 year, 2 years, and 5 years

Comparison of OKS of study subjects across follow-up				
OKS	Mean ± SD	Median (25th–75th percentile)	Range	*P* value
At 0 month	25.9 ± 8.1	26 (20–31)	7–46	< .0001*
At 6 months	38.4 ± 8.3	41 (33–45)	9–58	
At 12 months	40.1 ± 8.9	43 (36.3–46.8)	8–60	
At 24 months	41.8 ± 8.0	44 (39–47)	4–60	
At 60 months	42.4 ± 7.2	44 (40–47)	15–60	

Comparison of Tegner activity level of study subjects across follow-up				
Tegner activity level	Mean ± SD	Median (25th–75th percentile)	Range	*P* value
At 0 month	3.6 ± 1.6	3 (3–5)	0–7	0.001*
At 6 months	3.8 ± 1.5	4 (3–5)	1–6	
At 12 months	4.0 ± 1.4	4 (3–5)	0–6	
At 24 months	4.0 ± 1.4	4 (3–5)	1–6	
At 60 months	4.0 ± 1.8	4 (3–5)	1–7	

Comparison of VAS for pain of study subjects across follow-up				
VAS for pain	Mean ± SD	Median (25th–75th percentile)	Range	*P* value
At 0 month	6.0 ± 2.7	7 (4–8)	0–10	< .0001*
At 6 months	1.2 ± 1.5	1 (0–2)	0–8	
At 12 months	0.8 ± 1.2	0 (0–1)	0–9	
At 24 months	0.7 ± 1.4	0 (0–1)	0–9	
At 60 months	0.8 ± 1.4	0 (0–1)	0–9	

*Friedman test

**Table 4:** Comparison of OKS, Tegner activity level, and VAS for pain between 5 age subgroups at preoperatively and postoperatively 6 months, 1 year, 2 years, and 5 years

Comparison of OKS between age group					
OKS	50–59 (*n* = 5)	60–69 (*n* = 31)	70–79 (*n* = 86)	80–89 (*n* = 60)	*P* value
At 0 month	29 (28–38)	26 (17–30.5)	26 (21–30.8)	26 (20–31)	0.583^a^
At 6 months	43 (41–44)	41 (34.5–44)	41.5 (33.5–45)	38.5 (31–44)	0.342^a^
At 12 months	47 (46–47)	43 (37.5–46)	44 (36–47)	41 (36–46)	0.177^a^
At 24 months	46 (45–47)	44 (38–47)	45 (42–47)	43.5 (35.8–47)	0.428^a^
At 60 months	46 (45–48)	45 (39–47)	45 (42–47)	41.5 (38.8–46)	0.06^a^

Comparison of Tegner activity level between age group					
Tegner activity level	50–59 (*n* = 5)	60–69 (*n* = 31)	70–79 (*n* = 86)	80–89 (*n* = 60)	*P* value
At 0 month	6 (4–6)	3 (2–4)	4 (3–6)	3 (2–4)	0.019^a^
At 6 months	6 (5–6)	4 (3–5)	4 (3–5)	3 (2–4)	0.0008^a^
At 12 months	6 (4–6)	4 (3–4.5)	4 (3–6)	3 (3–4.3)	0.028^a^
At 24 months	5 (4–6)	4 (3–4.5)	4 (3–5)	3 (3–5)	0.078^a^
At 60 months	5 (4–6)	4 (3–4)	4 (3–6)	3 (3–4)	0.009^a^

Comparison of VAS for pain between age group					
VAS for pain	50–59 (*n* = 5)	60–69 (*n* = 31)	70–79 (*n* = 86)	80–89 (*n* = 60)	*P* value
At 0 month	5 (4–5)	7 (4–8)	7 (4–8)	7 (4–8)	0.754^a^
At 6 months	1 (1–1)	1 (0–2)	1 (0–2)	1 (0–2)	0.972^a^
At 12 months	0 (0–1)	1 (0–1)	0 (0–1)	1 (0–1)	0.192^a^
At 24 months	0 (0–0)	0 (0–1.5)	0 (0–1)	0 (0–1)	0.032^a^
At 60 months	0 (0–0)	0 (0–1)	0 (0–1)	0 (0–1.3)	0.633^a^

^a^Kruskal–Wallis test

Patients’ sports and physical activity improved significantly at 1 year compared to the preoperative activity level (*p* < 0.001). Although the Tegner activity level improved over time, this improvement was not significant (Table 3 and Fig. 2B) (NS). Physical activity was also analyzed for different age groups at different follow-up periods and no significant difference was found at 1 year and 2 years among the age groups (Table 4 and Fig. 2B) (NS). On comparing males and females, Tegner activity level is significantly higher in males than in females (Fig. 3B) (*p* < 0.004).

Significant improvement was found in the VAS for pain at all follow-up time points compared to the preoperative score (Table 3 and Fig. 1C) (*p* < 0.0001). A comparison of VAS for pain was performed at different follow-up periods and it was not significantly improved at 2-year and 5-year as compared to the score at 1-year (NS). On analyzing different age groups, VAS for pain significantly improved at 2-year follow-up in the different age groups (Table 4 and Fig. 2C) (*p* < 0.032). No significant difference was observed in the post-operative VAS for pain between male and female patients (Fig. 3C) (NS).

### Survivorship

None of the patients had any loosening of their implants or polyethylene wear and none of the knees was revised for any reason. All had been actively practicing their preferred sports until the last follow-up. Thus, survivorship was found to be 100% at 5 years of the minimum follow-up in this study.

## Discussion

The most important findings of this study are that sports participation improved significantly after TKA and the top three sports preferences in the summer session were hiking, cycling, and swimming, while in winter, they were skiing, cross-country skiing, and hiking. All the patients continued to practice sporting activities after surgery. OKS, Tegner activity levels, and VAS for pain also improved significantly from the preoperative level. Prosthesis survivorship was found to be 100% at the end of a 5-year follow-up.

It is increasingly recognized that younger people are also affected by osteoarthritis of the knee and these younger patients are typically more involved in sporting and physical activity; therefore, expectations after TKA are very high [1, 10]. Moreover, increasing life expectancy is leading to greater sports participation in older ages. Patients undergoing TKA expect to resume their sports activities after surgery and patients’ satisfaction is dependent on whether preoperative expectations are fulfilled [2].

In this study, sports participation improved significantly after TKA, and these findings are similar to the previous studies [9, 11]. In their meta-analysis, Konings et al. [11] reported that after TKA patients become more active in sports than before the onset of restricting symptoms. In another study [19] after a mean follow-up of 10 years following TKA, 169 (70.8%) patients stayed actively involved in sports, whereas in the current study at the end of the 5-year follow-up, all the patients continued to practice sporting activity. Of the 70.8%,71.3% of the patients continued practicing low-impact sports, the most common being swimming, hiking, Nordic walking, gymnastics, and fitness training. The type of sporting activity most common in their series is similar to the current study, accounting for regional differences. Plassard et al. [16] found that the mean time to return to sports was 5 months, and the most frequently performed sports were walking, hiking, gardening, swimming, yoga, cycling, and golf. Again, taking into account the cultural and regional differences, these findings are similar to the findings of this study in the most commonly practiced sports and return to sports after TKA. In a previous study [9] among the TKA patients, the most popular summer sports were hiking, cycling, and swimming. During the winter, the patients preferred skiing, cross-country skiing, and hiking, and no significant change in the preferred sports was observed among the patients after 2 years of follow-up. This mirrors the findings of the current study, at the end of our 5-year follow-ups.

Thaler et al. [18] performed an online survey of 120 European Knee Association (EKA) members. In their study, beyond 12 weeks after TKA, aerobics, hiking, and Nordic walking were reported as allowed, and after 6 months postoperatively, the survey participants additionally recommended mountain biking/incline cycling, and skiing sports (which were practiced by patients in the current study), highlighting that findings of this study are in line with the prevailing practice among European knee surgeons.

The OKS had improved overall at all points of time during the follow-up. The greatest improvement in OKS was obtained in the first 1 year after surgery, after which improvements, although seen, were not significant. Similar findings were reported by Hepperger et al. [9], who showed significant improvements in OKS after TKA in the first 6 months. It suggests that OKS significantly improved after TKA, but this improvement is seen during the first 12 months, and after that, no significant improvement is observed. Moreover, the OKS was not significantly correlated to age groups or gender in the current study, again similar to a previous study [9], where patients were categorized as young (72 and younger) and old (73 and older) and no significant difference was noted in OKS between the groups. This finding suggests that elderly patients can also be assured of a significant return of knee function, despite their advanced age and they can expect good outcomes after a TKA. Tegner activity levels were used as a measure of physical activity and sports participation of the patients, and it was found that they improved significantly at 1 year after TKA. Again, this finding is similar to the previous studies [9, 19], where the authors found that Tegner activity levels improved significantly at the end of their follow-up. The correlation between the different age groups and the Tegner activity level showed that younger groups had significant improvement compared to the older group. This suggests that age was a negative predictor of post-operative Tegner scores. Similar findings were also observed in other studies [9, 19]. This could be because elderly patients may be limited in their physical capacity due to other unrelated comorbidities and a younger and relatively more active patient’s knee would be in a better condition of muscle strength and balance [2]. The interesting fact which needs to be considered is that in the study by Vielgut et al. [19], the mean age of patients was 62.7 ± 11.4 years, while the current study has a mean patient age of 75.6 ± 7.2, and still, this study showed significant improvement in the Tegner activity level. The study showed that Tegner activity levels were significantly higher in the male patients than in females, which is in agreement with the findings of Hepperger et al. [9], and it indicates that after TKA male patients will have better sports activity than female patients.

Significant improvements in VAS scores were obtained in the first 12 months of the current study which is similar to the previous study [9]. It suggests that knee pain significantly improved after TKA, but this improvement is seen during the first 12 months, and after that, no significant improvement was observed. In addition, and again similar to findings by Hepperger et al. [9], there was no age- or gender-related correlation for VAS scores in this study.

While active sports participation has benefits for the patients’ overall health and quality of life, there always is a concern about implant loosening and accelerated polyethylene wear with greater exertion. Indeed, older literature suggests that sedentary and older patients had longer survivorship of their knee prostheses [7]. Some studies, however, have come out with different findings. In the prospective study by Mont et al. [15], two groups of 57 patients with an average age of 70 years were recruited according to the frequency and level of physical training. At an average of 7 years of follow-up, similar results were obtained relating to implant failure rate and X-ray evidence of loosening. In the current study, there was an implant survival rate of 100% at the end of the 60-month follow-up, with all the patients continuing their sporting activities. Similarly, in the previous study [19], aseptic implant loosening occurred in 3 patients, implant failure in 1 and polyethylene wear in 1 patient out of 236 patients at the end of a mean follow-up of 14.9 ± 3.0 years. Other recent studies also showed excellent survivorship of TKA [6, 17]. This demonstrates quite well that sporting activities do not negatively impact survivorship of the knee prosthesis and all of our patients are encouraged to take up sports participation after their TKA. This will not only provide a good outcome for the patient but also improve their quality of life.

A minimum follow-up of 5 years, data availability at all various points during follow-up and not just at start and endpoints, and stratification of patients into four age subgroups for analysis are the strengths of this study.

We would like to acknowledge the limitations which include the study being a retrospective analysis, the lack of a matched control group, and the use of only patient-reported outcome measures. The smaller sample size in the subgroup analysis is another limitation of the study. A prospective study with a large sample size should be conducted which will be of higher evidentiary value.

The findings of this study have clinical implications; when counseling the patient for TKA; the surgeon can advise the patient regarding the outcome and physical activity after surgery according to the patient's demographic status. Moreover, these findings can serve as a repository to use while informing patients on their long-term outcome prospects concerning pain relief, improvement of function, and the possibility of returning to or even taking up sporting activities for a healthy lifestyle.

## Conclusion

After TKA, patients can be able to return to sporting activity or even perform better than before surgery. Maximum improvement was noted in the first post-operative year. The male and younger groups perform better than the female and older groups. Sports and physical activity do not negatively impact survivorship of the knee prosthesis at mid-term follow-up and all patients are encouraged to take up sports participation after their TKA.
